# Oncological outcomes of high-grade T1 non-muscle-invasive bladder cancer treatment in octogenarians

**DOI:** 10.1007/s11255-021-02870-2

**Published:** 2021-04-26

**Authors:** Aleksander Ślusarczyk, Karolina Garbas, Piotr Zapała, Łukasz Zapała, Piotr Radziszewski

**Affiliations:** Department of General, Oncological and Functional Urology, Medical University of Warsaw, Lindleya 4, 02-005 Warsaw, Poland

**Keywords:** Non-muscle-invasive bladder cancer, BCG therapy, Elderly patients, Survival, Advanced age

## Abstract

**Purpose:**

To evaluate the outcomes of high-grade T1 non-muscle-invasive bladder cancer treatment (NMIBC) in elderly patients over 80 years of age.

**Methods:**

This is a retrospective single tertiary-centre study. Medical records of patients with T1 high-grade NMIBC treated with transurethral resection of the bladder tumour (TURBT) were reviewed. Among 269 patients with high-grade T1 NMIBC, 74 individuals were over 80 years of age at the time of surgery. Finally, 67 patients met the inclusion criteria.

**Results:**

Only 47.8% of patients (*N* = 32) received at least five of the six instillations of the BCG immunotherapy induction course. Oncological outcomes were compared between patients who received at least the induction course of BCG and non-BCG-treated patients matched to each other based on age and Charlson comorbidity index. Thirty case–control pairs were included in the final analysis. Rates of disease recurrence (80% vs. 53%) and cancer-specific mortality (40% vs. 10%) were significantly higher in the group of patients who did not receive BCG. BCG therapy, Charlson comorbidity index, haemoglobin concentration and the number of tumours > 3 in TURBT constituted independent prognostic factors for cancer-specific survival (CSS).

**Conclusion:**

BCG should be strongly recommended to patients with T1HG NMIBC despite advanced age and comorbidities. Already BCG induction improves CSS and reduces the recurrence rate in octogenarians with T1HG bladder cancer.

## Introduction

Bladder cancer is the eleventh most common malignancy worldwide [1]. In general, most patients present with non-muscle-invasive bladder cancer (NMIBC) regarded as tumour confined to the mucosa (Ta or carcinoma in situ) or the submucous layer of the urinary bladder wall (T1), which might be effectively managed with transurethral resection of the bladder tumour (TURBT) [1]. However, in NMIBC characterized as high-risk of progression (high-grade tumour, T1 category tumour or CIS), adjuvant treatment with intravesical Bacillus Calmette–Guerin (BCG) vaccine instillations is necessary to improve recurrence- (RFS) and progression-free survival (PFS) [2, 3]. High-grade T1 bladder tumour constitutes the most aggressive form of NMIBC with the highest risk for recurrence and progression [4, 5].

Patients’ age represents one of the greatest single risk factors for bladder cancer (BC) development [6]. The prevalence of bladder cancer is highest in an elderly population, with 73 years being an average age of diagnosis and peak incidence reported at 85 years old [7, 8]. The burden of BC in elderlies might be expected to grow as further ageing of the population is being observed [6]. BC patients over 80 years of age often present with several comorbidities, with cardiovascular diseases being the most frequent. Elderly patients require more individualized strategies to prevent disease progression without causing serious treatment complications and compromising their quality of life. Due to advanced age, comorbidities and disability, these patients are not always fitting or eager to obtain optimal recommended oncological treatment.

Relatively little is known regarding the efficacy of NMIBC treatment in elderly patients [9]. Several studies report a measurable impact of ageing on the efficacy of intravesical BCG therapy [10–12]. Results of recent reports suggest that the BCG therapy for NMIBC is less effective in patients > 70 years of age compared to younger ones [11]. More importantly, the study included only 20 patients aged 80 years and more, which was previously depicted as a threshold of particular BCG failure risk [10].

The potential complications of intravesical therapy might not be as well-tolerated in elderly individuals whose urinary, cardiovascular and immunological functions, as well as a pulmonary reserve, are decreased [6]. Some investigators caution against intravesical therapy in the elderly and suggest that due to complications, BCG maintenance should be avoided in the population > 80 years [6, 13].

Available studies encompassing a larger cohort of elderly NMIBC patients are lacking and guidelines do not recommend any treatment modification in that group. To date, subcohorts of large clinical trials provide limited insight into outcomes of BCG administration in patients older than 80 years [11].

In this study, we aimed to evaluate the results of T1HG bladder cancer treatment in patients over 80 years of age and assess the impact of intravesical BCG therapy on oncological outcomes.

## Methods

This is a single tertiary-centre case–control study. Medical records of patients who underwent transurethral resection of the bladder tumour in our department between 2010 and 2018 were retrospectively reviewed. Patients with T1 high-grade tumour in TURBT specimen and at least 80 years of age at the time of surgery were identified.

The surgeries were carried out according to the protocol recommended by the European Association of Urology (EAU) clinical guideline [1]. Surgical specimens were reviewed by a genitourinary pathologist, graded according to the 1973 and 2004 WHO grading system and staged according to the 2009 TNM classification.

Consecutive patients were treated with adjuvant BCG immunotherapy in accordance with EAU guidelines [1]. BCG induction included 6 weekly instillations, whereas the maintenance course consisted of 3 weekly instillations administered at 3, 6, 12, 18, 24, 30 and 36 months. Ninety-seven percent of patients (*N* = 31) were treated with RIVM BCG strain, with each dose including at least 2 × 10^8^ and no more than 3 × 10^9^ BCG. One patient (3%) was managed with Moreau strain, with each dose containing at least 3 × 10^8^ BCG. Patients always received a full dose of BCG.

Baseline characteristics including clinical, histopathological and laboratory data were collected. Histopathological data included staging and grading of each specimen. Clinical data included age, gender, previous history of bladder cancer, tumour size, multifocality and comorbidities. Each patient was retrospectively classified using Charlson comorbidity index (CCI) [14] and NMIBC dedicated prognostic tools (European Organization for Research and Treatment of Cancer (EORTC) risk tables and Spanish Urological Club for Oncological Treatment (CUETO) scoring model). Laboratory data included preoperative blood parameters—blood cell counts and haemoglobin concentration collected from pretreatment laboratory test.

Differences between groups were evaluated with the *U* Mann–Whitney test for continuous variables and Fischer’s exact test or chi-square test for categorical variables. Continuous variables are presented as median values accompanied by the interquartile range (IQR). For prediction analysis, univariable and multivariable Cox proportional hazard was used. The differences in time to event were evaluated with log-rank test and illustrated with Kaplan–Meier curves. Correlations between variables were checked using the Spearman test. For all statistical analyses, a two-sided *p* value < 0.05 was considered statistically significant. Statistical analysis was performed with the SAS System (version 9.4).

## Results

Among 269 patients with high-grade T1 non-muscle-invasive bladder cancer, 74 individuals were over 80 years of age at the time of surgery. Patients with T2HG at restaging TURBT (re-TURBT) (*N* = 4), patients without complete macroscopic resection (*N* = 2) and lack of follow-up (*N* = 1) were excluded from the analysis. Finally, sixty-seven patients met the inclusion criteria.

During a median of 29-month follow-up, 44 patients recurred and 26 patients developed progression. Only 47.8% of patients (*N* = 32) received at least five of six instillations of BCG immunotherapy induction course, whereas 29.9% of patients (*N* = 20) were treated with at least one maintenance BCG course. In the remaining 52.2% of patients (*N* = 35) who did not receive BCG, the most common causes were patient’s non-compliance (34.3%), presence of comorbidities including uncontrolled systemic diseases (17.1%), and guidelines non-adherence due to suspected non-compliance (14.3%). Other less frequent causes were BCG intolerance (5.7%), dementia (5.7%), mobility impairment (5.7%), delay in BCG initiation (2.9%) and unknown reason (8.6%).

Oncological outcomes were compared between patients who received at least the induction course of BCG and non-BCG-treated patients matched to each other based on age and Charlson comorbidity index. Patients selected after case–control matching included 30 BCG-treated and 30 non-treated individuals, as presented in Table 1. Further analysis was performed on a cohort of 60 case–control matched patients, in whom 40 recurrences and 24 progressions were observed.

**Table 1:** Descriptive characteristics of patients with T1HG bladder cancer over 80 years at the time of surgery

	Overall		Non-BCG group		BCG group		*p* value
	No. Pts./Median	%/IQR	No. Pts./Median	%/IQR	No. Pts./Median	%/IQR	
Characteristics							
Male gender	50	83.3	22	73.3	28	86.7	NS
Grade 3	50	83.3	22	73.3	28	93.3	0.08
Concomitant CIS	7	11.7	1	3.3	6	20	**0.047**
Recurrent tumour	24	40	11	36.7	13	43.3	NS
Multiple tumours	35	58.3	20	66.7	15	50	NS
More than three tumours	19	31.7	9	30	10	33.3	NS
Big tumour ≥ 3 cm	27	45	16	53.3	11	36.7	NS
No muscle in TURBT specimen	9	15	5	16.7	4	13.3	NS
HG tumour history	15	25	9	30	6	20	NS
ReTURBT performance	43	71.7	20	66.7	23	76.7	NS
Tumour in reTURBT	22	36.7	8	40	14	60.9	0.18
HG tumour in reTURBT	25	41.7	8	25	17	56.5	**0.037**
Comorbidities							
Hypertension	49	81.7	26	86.7	23	76.7	NS
Coronary artery disease	39	65	15	50	24	80	**0.014**
Myocardial infarction	12	20	4	13.3	8	26.7	NS
COPD	6	10	3	10	3	10	NS
Diabetes mellitus type 2	17	28.3	10	33.3	7	23.3	NS
Dementia	2	3.3	2	6.7	0	0	0.15
History of other cancer	11	18.3	2	6.7	9	30	**0.020**
Stroke	17	28.3	9	30.5	8	25.4	NS
Atrial fibrillation	14	23.3	6	20	8	26.7	NS
Heart failure	17	28.3	8	26.7	9	30	NS
UTUC	3	5	2	6.7	1	3.3	NS
Other							
Age	85	82–89	85	81–89.5	85	82.5–88	NS
Charlson comorbidity index	7	6–8	7	6–8	7	6–8	NS
ASA	3	3–3	3	3–3	3	3–3	NS
Haemoglobin (g/L)	128	116–141.5	129.5	113–143	127	119–139	NS
CUETO recurrence score	8	5–10	7.5	5–10	8	5–11	NS
CUETO progression score	9	8–10	8	8–10	9	8–10	0.09
EORTC recurrence score	8	5–9	8	6–9	7	5–8	NS
EORTC progression score	14	9.5–15	14	9–15	14	11–15	NS
Outcomes							
Recurrence	40	66.7	24	80	16	53.3	**0.029**
Progression	24	40	15	50	9	30	0.11
All-cause death	33	55	20	66.7	13	43.3	0.07
Cancer-specific death	15	25	12	40	3	10	**0.007**

Comparison between BCG-treated (*N* = 30) and non-treated groups (*N* = 30)

*CIS* carcinoma in situ, *HG* high-grade, *reTURBT* restaging transurethral resection of bladder tumour, *COPD* chronic obstructive pulmonary disease, *ASA* American Society of Anesthesiologists Score, *IQR* interquartile range, The bold values denote statistical significance at the p < 0.05 level

The presence of concomitant CIS (*p* = 0.047), residual high-grade tumour in re-TURBT (*p* = 0.037), history of other cancer (*p* = 0.020) and coronary artery disease (*p* = 0.014) were more frequent in the BCG-treated cohort. Both groups did not differ significantly in terms of other major factors that could influence oncological outcomes and overall survival.

Rates of disease recurrence (80% vs 53%) and cancer-specific mortality (40% vs 10%) were significantly higher in the group of patients who did not receive BCG compared to BCG-treated ones.

**Fig. 1:**
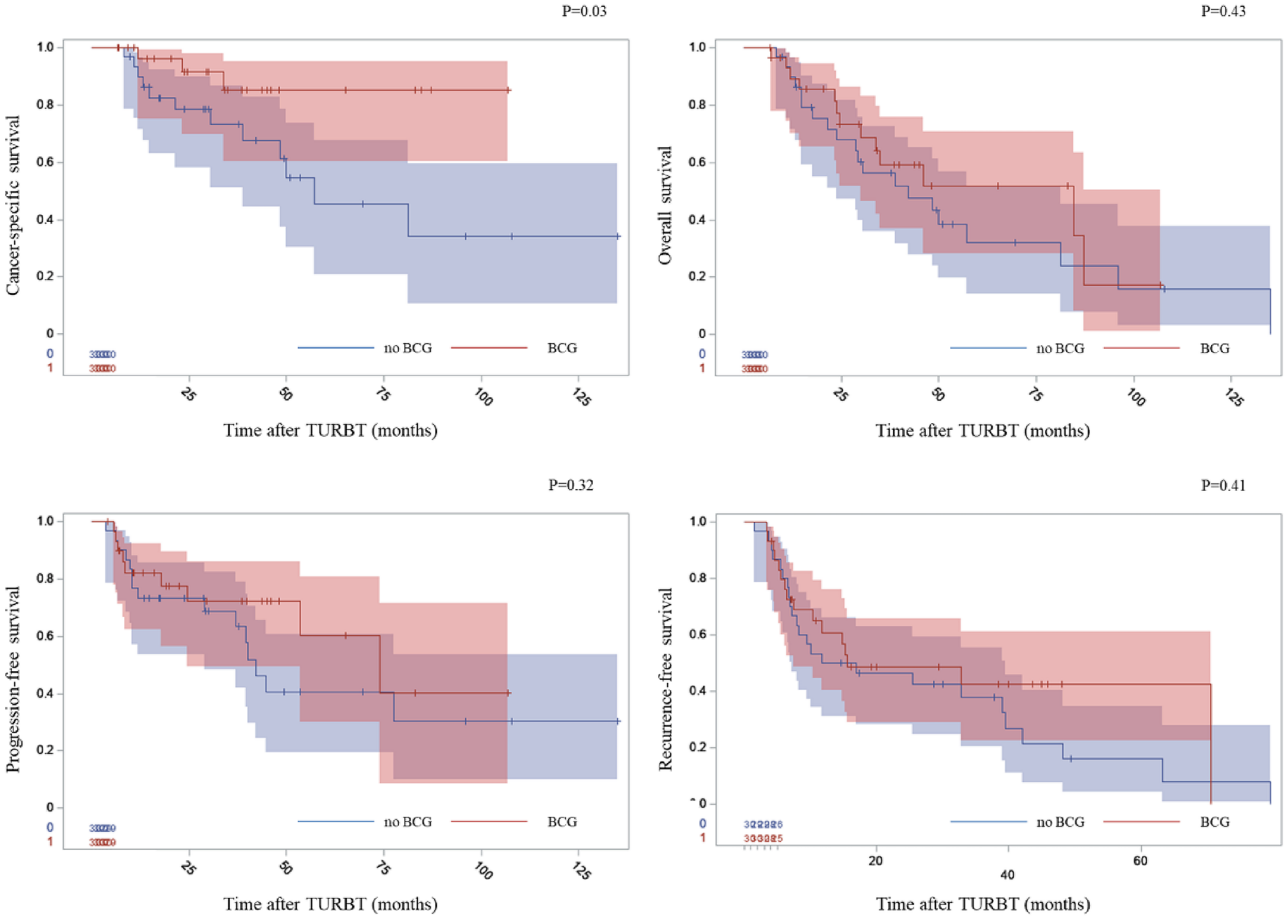
Kaplan–Meier curves representing the influence of induction BCG therapy on oncological outcomes and overall survival

In Kaplan–Meier analysis, BCG induction was associated with favourable cancer-specific survival (CSS) (Fig. 1). We did not observe the association between BCG induction course and the time to recurrence or progression, though (Fig. 1). However, the higher number of received BCG courses was associated with better RFS in multivariate analysis. EORTC or CUETO risk scores did not predict the RFS or PFS in our study population. Univariate analysis for CSS was performed (see Table 2 for details). Cox proportional hazards were utilized to investigate which factors are independently associated with RFS, PFS, CSS and overall survival (OS) (see Table 3 for details). There were no correlations between the variables included in each model.

**Table 2:** Univariate Cox proportional hazard regression analysis for prediction of cancer-specific survival in T1HG patients > 80 years

Clinicopathological features		HR	95% CI	*p*
Age		1.046	0.92–1.19	NS
BCG induction	(Yes vs no)	0.271	0.08–0.96	**0.044**
Number of BCG courses		0.514	0.26–1.04	**0.062**
Male gender	(Yes vs no)	0.350	0.12–1.03	**0.057**
Grade (yes vs no)	(3 vs 2)	4.389	0.56–34.08	0.16
Concomitant CIS	(Yes vs no)	0.602	0.08–4.73	NS
Recurrent tumour	(Yes vs no)	0.994	0.35–2.85	NS
Multiple tumours	(Yes vs no)	1.724	0.59–5.08	NS
Tumour size ≥ 3 cm	(Yes vs no)	2.240	0.79–6.34	0.13
Muscle in TURBT specimen	(No vs yes)	2.998	0.95–9.48	**0.062**
No. of tumours	(> 3 vs ≤ 3)	2.409	0.81–7.14	0.11
HG tumour history	(Yes vs no)	1.381	0.47–4.07	NS
reTURBT	(Yes vs no)	0.635	0.22–1.87	NS
Tumour in reTURBT	(Yes vs no)	1.757	0.48–6.48	NS
HG tumour in reTURBT	(Yes vs no)	2.031	0.57–7.23	NS
Comorbidities				
Hypertension	(Yes vs no)	5.445	0.70–42.11	0.10
Coronary artery disease	(Yes vs no)	0.661	0.24–1.84	NS
Myocardial infarction	(Yes vs no)	1.188	0.33–4.25	NS
COPD	(Yes vs no)	1.765	0.60–5.18	NS
Diabetes mellitus type 2	(Yes vs no)	0.833	0.27–2.62	NS
History of other cancer	(Yes vs no)	1.020	0.29–3.63	NS
Atrial fibrillation	(Yes vs no)	0.631	0.18–2.24	NS
Heart failure	(Yes vs no)	1.765	0.60–5.12	NS
UTUC	(Yes vs no)	5.445	0.70–42.11	0.10
Other				
Charlson comorbidity index		1.514	0.98–2.35	**0.064**
ASA		1.104	0.56–2.18	NS
Haemoglobin		0.976	0.95–1.00	**0.089**
NLR		1.080	0.88–1.33	NS
CUETO recurrence score		1.119	0.95–1.31	0.17
CUETO progression score		1.186	0.86–1.63	NS
EORTC recurrence score		1.207	1.00–1.46	**0.051**
EORTC progression score		1.095	0.96–1.25	NS

*HR* hazard ratio, *CI* confidence interval, *CIS* carcinoma in situ, *reTURBT* restaging transurethral resection of bladder tumour, *COPD* chronic obstructive pulmonary disease, *UTUC* upper tract urothelial cancer, *ASA* American Society of Anaesthesiologists Score, *NLR* neutrophil-to-lymphocyte ratio, The bold values indicate p < 0.1 values

**Table 3:** Multivariate Cox proportional hazard regression analysis for prediction of (1) cancer-specific survival, (2) overall survival, (3) progression-free survival and (4) recurrence-free survival in T1HG patients > 80 years

	Multivariate analysis		
	HR	95% CI	*p*
CSS			
BCG induction therapy	0.234	0.06–0.87	0.030
No BCG therapy	Reference		
Charlson comorbidity index	1.711	1.04–2.82	0.035
Haemoglobin	0.962	0.96–0.93	0.014
No. of tumours > 3	3.173	1.00–10.09	0.050
No. of tumours ≤ 3	Reference		
OS			
Multiple tumours	2.979	1.32–6.75	0.009
Single tumour	Reference		
Charlson comorbidity index	1.380	0.97–1.96	0.074
Haemoglobin	0.964	0.94–0.98	0.0004
PFS			
Tumour size ≥ 3 cm	2.387	1.03–5.53	0.042
Tumour size < 3 cm	Reference		
Charlson comorbidity index	1.579	1.09–2.28	0.015
RFS			
BCG—number of courses	0.776	0.61–0.99	0.038
reTURBT			
Not performed	3.720	1.52–9.09	0.004
Residual tumour	2.829	6.54–1.23	0.015
No residual tumour	Reference		

*HR* hazard ratio, *CI* confidence interval, *reTURBT* restaging transurethral resection of bladder tumour

Clinically significant side effects of BCG administration were observed in 32% of patients (*N* = 11). This included sudden cardiac arrest (*N* = 1), systemic side effects such as inflammatory response (*N* = 2) and local side effects (*N* = 8). BCG intolerance, regarded as withdrawal due to severe side effects, occurred in six patients.

## Discussion

In our study, we analysed the oncological outcomes of T1HG NMIBC in patients older than 80 years at the time of TURBT. We aimed to identify factors associated with compromised cancer-specific survival in the geriatric population of T1HG patients. Cases were matched to controls based on age and comorbidities, leading to the inclusion of 30 BCG-treated and 30 non-treated patients into the final analysis.

Intravesical BCG therapy, low Charlson comorbidity index, higher haemoglobin concentration and three or fewer tumours in TURBT were associated with better cancer-specific survival. Haemoglobin was previously reported as OS and CSS predictor in the cohort of NMIBC patients [15, 16], and our study validates its prognostic role in elderly T1HG individuals. Our observations underline the pivotal role of BCG treatment and provide evidence that BCG administration is associated with prolonged CSS in the group of elderly > 80 years. Suboptimal BCG use in elderly patients remains a significant problem observed not only in our study [17, 18]. It has been already reported that in the large cohort of 23,932 eligible NMIBC patients above 65 years, only 22% received adjuvant BCG [17]. In the same study, only 39% of patients with stage CIS or T1 received BCG. In a Swedish cohort of patients with T1 pathology, only 24% were treated with BCG [19]. Moreover, another study demonstrated that among 1590 patients who underwent a single resection of high-grade tumour 59.6% did not receive any BCG therapy, whereas 25.8% received only partial induction. Multiple resections and younger chronological age, but not the presence of comorbidities, were associated with BCG initiation [18]. In several studies, age > 80 years was found to increase the risk of not receiving BCG [17, 18]. Unexpectedly, in our study encompassing only patients older than 80 years, neither age nor the presence of comorbidities were related to BCG administration. Residual high-grade tumour at restaging TURBT and concomitant carcinoma in situ were associated with receiving at least BCG induction course.

BCG immunotherapy remains a gold standard therapy, which prevents tumour recurrence and progression [2, 3]. Although some evidence suggested a lower efficacy of BCG in an older subgroup of patients [10–12], guidelines strongly recommend BCG initiation regardless of age and comorbidities [1]. Till date, the survival benefit from BCG therapy is not as well evidenced as its efficacy in preventing recurrence and progression [2, 3, 20]. Our analysis confirms the CSS benefit from receiving at least BCG induction, which underlies the importance of BCG initiation in high-risk patients regardless of age. Moreover, in our study, patients treated with BCG compared to the control group presented more frequently with concomitant CIS and had higher rates of residual high-grade tumour in re-TURBT and still achieved better CSS.

The recurrence rate was significantly lower (80% vs 53%; *p* = 0.03) and the rate of progression (50% vs 30%; *p* = 0.11) seemed to be lower in the BCG-treated group, as the relationship tended to statistical significance. Residual tumour at re-TURBT, lack of re-TURBT performance and number of received BCG courses constituted independent predictors of RFS, whereas tumour size ≥ 3 cm and presence of comorbidities (assessed using the Charlson comorbidity index) were associated with PFS. This emphasizes the fundamental role of BCG administration in preventing disease recurrence and indicates that big tumours (≥ 3 cm) are characterized by the increased potential to infiltrate the muscle layer of the urinary bladder wall. Big tumour size was already shown as an independent prognostic factor for PFS in the T1HG BC population [21]. Inclusion of re-TURBT performance as an independent predictor of RFS underlies its substantial role in high-risk NMIBC treatment, even though it was not associated with CSS benefit. Re-TURBT is therefore a crucial procedure for therapeutic and staging purposes [22, 23].

Notably, 79% of patients who progressed to muscle-invasive disease and were dead at the last follow-up died from bladder cancer. Such observation might be attributed to the frequent presence of cardiovascular diseases and other comorbidities in the studied population, which were the actual causes of death in the remaining 21% of patients. Independent factors associated with overall survival were the following: Charlson comorbidity index, haemoglobin concentration and the presence of multiple tumours. In the Kaplan–Meier analysis, we did not observe overall survival benefit related to BCG use, but this might result from a small sample size. On the other hand, a small but significant survival advantage associated with BCG in the elderlies was reported by Spencer et al. [17].

As there was no correlation between the Charlson comorbidity index and BCG administration, CCI appeared to be an independent prognostic factor for oncological outcomes (PFS and CSS) and, unsurprisingly, for overall survival. Therefore, routine CCI estimation might be of clinical use in geriatric bladder cancer patients to guide suitable individualized therapy and follow-up. It has been already reported that CCI might be utilized as a predictor of radical cystectomy outcomes [24]. The impact of comorbidities on treatment effectiveness and compromised compliance among cancer patients has already been summarized by Søgaard et al. [25].

In the present study, we observed suboptimal BCG use with less than half of patients receiving at least induction BCG therapy. Efforts should be made to increase the rate of BCG receivers among elderlies with high-risk NMIBC and improve patient’s compliance without the quality of life impairment, which might be even a greater challenge in the era of Covid-19 pandemic.

## Conclusion

In our study, we confirm the utmost importance of BCG therapy initiation in elderly high-risk NMIBC patients. To conclude, BCG should be strongly recommended to patients with T1HG despite advanced age and comorbidities. Already BCG induction improves cancer-specific survival and reduces the recurrence rate in T1HG patients over 80 years of age.

## Study limitations

Limitations of the study result from its retrospective design. The follow-up was inconsistent among patients and the sample size was small. Lack of significant association between RFS, PFS and BCG use might be attributed to the cohort size and relatively short, inconsistent follow-up in many cases. Censored data at the time of the last follow-up and a wide 95% confidence interval might also contribute to the lack of statistically significant PFS differences.
